# Hospitals and Payment for Wounded

**Published:** 1915-03-27

**Authors:** Conrad W. Thies

**Affiliations:** Hon. Sec., British Hospitals Association.


### THE EDITOR'S LETTER-BOX.

### Hospitals and Payment for Wounded.

*To the Editor of* The Hospital.

Sir,—The question which was recently asked in the House of Commons by Mr. H. Lawson, as to what was the basis upon which contributions were made to those voluntary hospitals which had admitted sick and wounded soldiers, was answered by Mr. Baker to the effect that the use of these hospitals had not been offered by their managers on a commercial basis, and that the assistance given to these institutions by the Government had taken the form of grants in aid on the basis of a daily rate ranging from 2s. to 4s. for each occupied bed. Replying to a further question as to whether any difference had been made between the voluntary hospitals of London and those of the provinces, Mr. Baker stated "that he could not answer this question."

In these circumstances it is desirable that the facts should be made public. Immediately upon the outbreak of war most of the voluntary hospitals offered the use of beds for wounded soldiers and sailors, leaving it entirely to the Government to decide if any financial assistance would be rendered to these institutions.

From inquiries which were instituted by the British Hospitals Association, it was ascertained that the arrangements which had been made with the hospitals as to payment varied very considerably. For instance, few, if any, of the London hospitals received any remuneration for their services, while most of the provincial hospitals had arranged to be paid at rates varying from 2s. to 4s. for each soldier patient per day.

Although, influenced by patriotic motives, the managers of the London hospitals did not in the first instance ask for payment, they now, with few exceptions, express a desire to receive payment. In some cases where this question was raised the managers were definitely informed that if payment was asked for no soldier patients would be sent.

It will be generally agreed that it is the duty of the Government to secure the most efficient treatment possible for those who have been wounded in the service of their country. The cost of such treatment should be borne by the State, and not be made a charge upon the financial resources of the voluntary hospitals, especially as at the present time these charitable institutions find increasing difficulty in obtaining funds to carry on their work, the cost of which has been so greatly increased by the war.

It must be borne in mind that the voluntary hospitals are able to provide the best medical and surgical treatment for soldiers, in consequence of the exceptional skill and experience of their medical, surgical, and nursing staffs and the completeness of their equipment. Up to the present time the resources of these institutions have not been fully utilised; a large proportion of the soldier patients who have been sent to them for treatment suffering only from slight wounds or ailments could have been equally well treated in the "voluntary aid hospitals" or convalescent homes.

In these circumstances, the Council of the British Hospitals Association made an application to the War Office, on behalf of the hospitals, requesting that all the voluntary hospitals who received wounded soldiers should be remunerated upon some equitable, although not necessarily uniform, scale.

A reply was received to the following effect:

That the general principle adopted as to payment for hospital accommodation for wounded soldiers is that the hospital concerned should take up the question with the general officers of the command or district in which the hospital is situated, with a view to fixing a reasonable rate of payment, having regard to the circumstances of each case. It will be obvious that considerable variation in the rates paid is inevitable in the circumstances, but the Council are of opinion that the system adopted is that best suited to meet the needs of the situation, and has, so far as they know, worked satisfactorily hitherto.

The Council of the British Hospitals Association have accordingly recommended all the voluntary hospitals who have not already made arrangements for payment, but who desire payment to be made for soldier patients, to apply to the local military authorities as suggested by the War Office.—Yours faithfully,

Conrad W. Thies,
Hon. Sec., British Hospitals Association.

